# Mechanistic Insights into the Ameliorative Effect of Cichoriin on Diabetic Rats—Assisted with an In Silico Approach

**DOI:** 10.3390/molecules27217192

**Published:** 2022-10-24

**Authors:** Hany Ezzat Khalil, Miada F. Abdelwahab, Hairul-Islam Mohamed Ibrahim, Khalid A. AlYahya, Ahmed Adel Mohamed, Amira Samir Radwan, Shaimaa Waz

**Affiliations:** 1 Department of Pharmaceutical Sciences, College of Clinical Pharmacy, King Faisal University, Al-Ahsa 31982, Saudi Arabia; 2 Department of Pharmacognosy, Faculty of Pharmacy, Minia University, Minia 61519, Egypt; 3 Department of Biological Sciences, College of Science, King Faisal University, Al-Ahsa 31982, Saudi Arabia; 4 Pondicherry Centre for Biological Science and Educational Trust, Puducherry 605004, India; 5 Department of Surgery, College of Medicine, King Faisal University, Al-Ahsa 36363, Saudi Arabia; 6 Department of Pharmacy Practice, College of Clinical Pharmacy, King Faisal University, Al-Ahsa 31982, Saudi Arabia; 7 Department of Biochemistry, Faculty of Pharmacy, Minia University, El-Minia 61511, Egypt

**Keywords:** cichoriin, streptozotocin, diabetes, SOD, GLUT4, AMPK, PI3K

## Abstract

Type 2 diabetes mellitus is considered to be a substantial socioeconomic burden worldwide on both patients and governments. Coumarins are biomolecules with a diversity of biological activities. The current investigation aimed to explore the ameliorative effects of cichoriin, which is a type of coumarin, on high-fat diet/streptozotocin (HFD/STZ)-induced diabetic rats. Methods: Rats were allocated into five groups. Group I was considered as the control group, while the other groups were HFD/STZ-induced diabetic rats. Group II was assigned as the diabetic control. Groups III and IV were treated with cichoriin (50 or 100 mg/kg, respectively). Group V received glibenclamide (5 mg/kg) (as a positive control). The blood glucose (BG), serum insulin, triglycerides (TG), total cholesterol (TC), total antioxidant capacity (TAC), catalase, hepatic superoxide dismutase (SOD) and content of malondialdehyde (MDA) were assessed. Histopathological and immunohistochemistry analysis of pancreatic tissue were performed. mRNA and protein expressions of GLUT4, AMPK, and PI3K were estimated. Results: Cichoriin treatment ameliorated HFD/STZ-induced diabetic conditions and mitigated the histopathological characteristics of the pancreas, as well as increasing pancreatic insulin expression. This decreased the levels of BG, TG, TC, and MDA and improved the TAC, catalase and SOD contents. Cichoriin demonstrated upregulation of mRNA and protein expressions of GLUT4, AMPK, and PI3K. The in silico binding of cichoriin with GLUT4, AMPK, and PI3K supported the possible current activities. Conclusion: Collectively, this work highlighted the potential role of cichoriin in mitigating HFD/STZ-induced diabetic conditions and showed it to be a valuable product.

## 1. Introduction

Diabetes mellitus (DM) is a chronic endocrinological impairment identified by persistent hyperglycemia [1]. The incidences of DM have increased by about four times in the last 35 years [2]. The number of diabetic patients reached more than 422 million. The reported cases are expected to rise to 592 million by the year 2035 [3]. In 2019, DM was considered the ninth leading cause of death because of the associated life-threating complications, including cardiovascular, cerebrovascular and renal disorders [4]. Type 2 DM is the most common type of DM, representing approximately 95% of all cases [1]. The therapy requires glucose-lowering agents that comprise insulin secretagouges (e.g., sulfonylureas), insulin sensitizers (e.g., thiazolidinediones and metformin) and glucosidase inhibitors (e.g., miglitol). Most of these medications produce undesirable effects, such as hypoglycemia, hepatic damage, lactic acidosis and neurological disorders [5]. Accordingly, researchers are striving to discover more antidiabetic medications, aiming to manage and expand the therapeutic strategy of the disease with safe and effective entities [6,7]. Herbs and herbal-derived drugs are estimated to be an important defense line against numerous illnesses and their complications [8,9]. Recent reports highlighted and summarized the common natural products that have been extensively employed to control blood glucose levels in diabetic patients [4,10,11,12]. Coumarins and their derivatives, as examples of bioactive metabolites, contributed to drug research and development owing to their multiple targets, varied biological activities and safety [13]. They have been shown to exhibit antioxidant activity by inhibiting lipid peroxidation [14], antibacterial effects against a variety of Gram-positive, Gram-negative and multidrug-resistant bacteria [15]; anticoagulant [16], anti-inflammatory [17], and anticancer effects against various cancer cell lines (including HeLa, MCF-7, MBA-231 and HepG2) [18]; and enzyme inhibition (such as glucosidase, aldose reductase, urease, monoamine oxidase and alkaline phosphatase inhibitors) [19] and antiviral (e.g., influenza and HIV) [20] activities. Interestingly, various coumarins and their derivatives demonstrated hypoglycemic activity acting on diverse targets for diabetes treatment, including the improvement of pancreatic function by reducing oxidative stress and inflammation, initiating insulin secretion, or stimulating the effect of insulin on its target peripheral organs. Some coumarins activate phosphatidylinositol-3-kinase (PI3K) and/or 5′-AMP-activated kinase (AMPK) as the main signaling pathways that promote glucose transporter type 4 (GLUT4) expression, which mediates glucose transport in fat and muscle and improves insulin sensitivity [21,22]. Cichoriin (6-hydroxy-7-O-glucosylcoumarin or aesculetin 7-glucoside) is a member of the coumarins with an attached sugar moiety that enhances its water solubility, bioavailability and oxidative stability with reduced toxicity [23]. Cichoriin has been shown to display interesting bioactivities, namely antibacterial [24], antifungal [25], antiviral [26] antioxidant [27], anti-inflammatory [28] and photoprotective activities [29]. Moreover, cichoriin was indicated to have average synthetic accessibility; therefore, it could be readily obtained and may be assessed for many targets. In view of the above, the current study highlights the in vivo evaluation of the antidiabetic activity of cichoriin via the histopathological observation of pancreatic tissue using a combination of high-fat diet (HFD) with streptozotocin (STZ)-induced diabetic rats, as well as the exploration of the involved molecular mechanism in the treatment of type 2 DM. The results were also supported by in silico studies and compared with those of the reference antidiabetic drug glibenclamide (GB). This is the first report of cichoriin as a potential natural antidiabetic candidate that could ameliorate DM.

## 2. Results

### 2.1. Effect of Cichoriin on Blood Glucose Level and Serum Insulin of Diabetic Rats

Based on the results shown in Table 1, the blood glucose level (BGL) was significantly increased (*p* < 0.05) in group II (diabetic) compared to the control. Interestingly, cichoriin at both doses (50 and 100 mg/kg) could significantly decrease (*p* ≤ 0.05) BGL compared to the diabetic group, with a non-significant difference from group V (reference drug, GB). The diabetic group (II) demonstrated a significant decrease (*p* ≤ 0.05) in serum insulin compared to that of the control group (I). Alternatively, cichoriin administration (group III and IV) significantly increased (*p* ≤ 0.05) the serum insulin level compared to the diabetic group. A high dose (100 mg/kg) showed a higher improvement effect than the dose of 50 mg/kg, with a similar insulin level to that of GB (group V).

### 2.2. Effect of Cichoriin on Triglycerides and Total Cholesterol 

A combination of HFD with STZ (group II) caused a significant elevation (*p <* 0.05) of triglycerides (TG) and total cholesterol (TC) levels, as compared to that of the control group. A low dose of cichorin (50 mg/kg) reduced TG and TC, but could not significantly decrease the elevated lipid profile when compared to the diabetic group. However, the high dose (100 mg/kg) revealed a significant reduction (*p* ≤ 0.05) in TG and TC in comparison to the diabetic group and exhibited better improvement than that of the reference group (Table 1).

### 2.3. Effect of Cichoriin on Oxidative Stress Markers

Table 2 illustrated a significant decrease (*p* < 0.05) in serum total antioxidant capacity (TAC), catalase and hepatic antioxidant superoxide dismutase (SOD) activities, as well as a significant increase in the malondialdehyde (MDA) level that occurred in the diabetic group compared to that of the control. Alternatively, the administration of cichoriin (50 mg/kg) normalized SOD and MDA levels, but this result was still non-significant in the diabetic group. However, cichoriin (100 mg/kg) caused a significant improvement (*p* ≤ 0.05) in TAC, catalase, SOD, and MDA in comparison to the diabetic group and similar to that of the reference group (5 mg/kg GB).

### 2.4. Histopathological Analysis of Pancreatic Tissue

**Figure 1:**
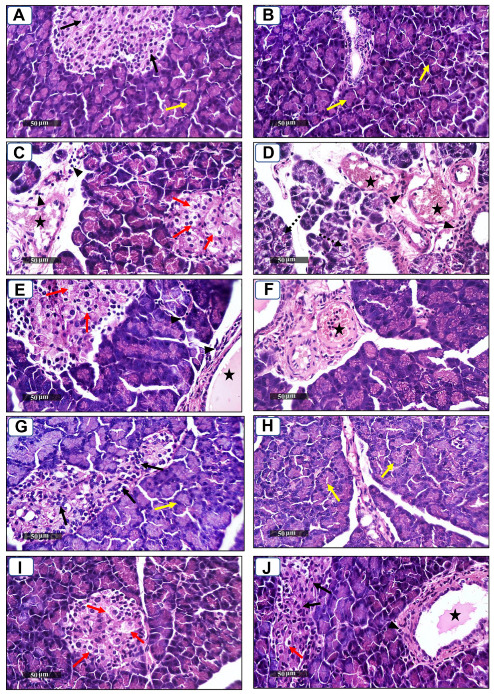
Photomicrograph sections of pancreases from control and diabetic rats with or without treatments. Group I (control) (**A**,**B**) showed normal organized pancreatic parenchyma with apparent intact pancreatic islets (black arrow), intact pancreatic acini (yellow arrow); group II (diabetic) (**C**,**D**) showed moderate swelling and vacuolar degenerative changes in pancreatic islets beta cells (red arrow), marked congested and dilated interlobular blood vessels (star). Mild records of perivascular inflammatory cells (arrow head) were shown with focal records of degenerated pancreatic acini (dashed arrow); group III (50 mg/kg, cichoriin) (**E**,**F**) showed almost the same persistent records as diabetic samples, group IV (100 mg/kg, cichoriin) (**G**,**H**) showed minimal records of cellular swelling and degenerative changes all over pancreatic islets (black arrow) or acini (yellow arrow), and group V (5 mg/kg, GB)) (**I**,**J**) showed persistent records of degenerative changes of some pancreatic islets (red arrow) alternated with almost apparent intact islets with intact subcellular details (black arrow), and congested/dilated BVs (star) with mild inflammatory cells infiltrates were shown (arrow head). Scale bar = 50 μm (400× magnification).

Group I (control) (Figure 1A,B) demonstrated normal organized morphological features of pancreatic parenchyma with many apparent intact pancreatic islets with normal subcellular structures (black arrow), intact pancreatic acini (yellow arrow) with intact interlobular scanty connective tissue with intact vasculatures. Group II (diabetic) (Figure 1C,D) demonstrated moderate swelling and vacuolar degenerative changes in pancreatic islets beta cells (red arrow) accompanied with marked congested and dilated interlobular blood vessels (star). Mild records of perivascular inflammatory cells (arrowhead) were shown with focal records of degenerated pancreatic acini (dashed arrow). Group III (50 mg/kg, cichoriin) (Figure 1E,F) showed almost the same persistent records as diabetic samples. Group IV (100 mg/kg, cichoriin) (Figure 1G,H) showed more significant protective efficacy compared to group III (50 mg/kg, cichoriin) with minimal records of cellular swelling and degenerative changes over all the pancreatic islets (black arrow) or acini (yellow arrow) with normal interlobular septa showing normal vasculatures as well as minimal cellular infiltrates. Group V (5 mg/kg, GB) (Figure 1I,J) showed persistent records of degenerative changes in some pancreatic islets (red arrow) alternated with almost apparent intact islets with intact subcellular details (black arrow); however, persistent records of congested/dilated BVs (star) with mild inflammatory cells infiltrates were shown (arrowhead).

### 2.5. Effect of Cichoriin on Pancreatic Insulin Immunoexpression in Diabetic Rats

**Figure 2:**
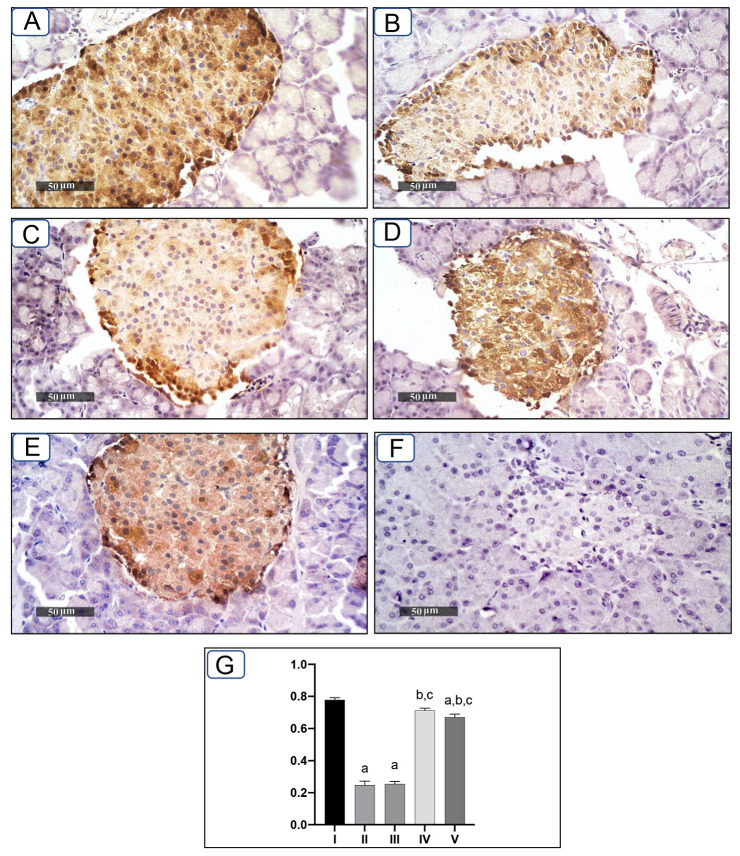
Immunohistochemical expression of insulin in pancreatic tissues in different groups. Group I (control) (**A**), group II (diabetic) (**B**), group III (50 mg/kg, cichoriin) (**C**), group IV (100 mg/kg, cichoriin) (**D**), group V (5 mg/kg, GB) (**E**), negative control (**F**), and semi-quantitative analysis of immunoreactivity of anti-insulin antibody in stained pancreatic sections of different groups (**G**). Data are represented as mean optical density ± SE. ^a^, ^b^, and ^c^ indicate significant difference from group I, group II, and group III, respectively, at (*p* ≤ 0.05).

As shown in Figure 2, pancreatic tissue sections of rats in the control group exhibited strong positive immunoreactivity of pancreatic islets against insulin antibody (Figure 2A); however, pancreatic sections from the diabetic group showed a significant reduction in insulin immunoreactivity in pancreatic islets compared to that of the control (Figure 2B). While cichoriin administration at a dose of 50 mg/kg did not improve the pancreatic expression of insulin in diabetic rats, a dose of 100 mg/kg showed a significant elevation in the pancreatic expression of insulin when compared to that of the diabetic group, and a similar effect to that of the reference drug (GB). Interestingly, cichoriin (100 mg/kg) restored the insulin expression to that of the control group.

### 2.6. Effect of Cichoriin on mRNA and Protein Expression of GLUT4, AMPK, and PI3K in Skeletal Muscle of Diabetic Rats

**Figure 3:**
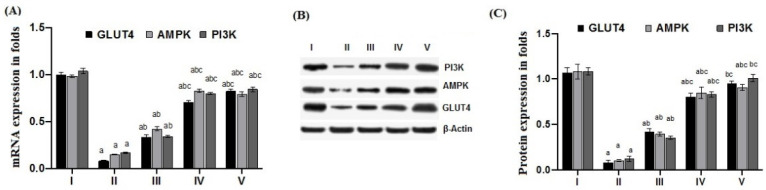
Effect of cichoriin on gene and protein expression of insulin sensitizing proteins in diabetic rats. Quantitative genes expression of GLUT4, AMPK, and PI3K (**A**). Quantitative analysis of affected proteins: GLUT4, AMPK, and PI3K (**B**,**C**). Group I (control), group II (Diabetic), group III (50 mg/kg, cichoriin), group IV (100 mg/kg, cichoriin), and group V (5 mg/kg, GB). a, b, c indicate significant difference from group I, group II and group III, respectively, at (*p* ≤ 0.05). Glucose transporter type 4 (GLUT4), AMP-activated protein kinase (AMPK) and phosphatidylinositol 3-kinase (PI3K).

The induction of diabetes with HFD/STZ caused a significant inhibition (*p* ≤ 0.05) of genes and proteins expression (Figure 3) of all studied insulin-sensitizing signaling pathways; GLUT4, AMPK, and PI3K in skeletal muscle when compared to that of the control. However, cichoriin administration showed a significant elevation (*p* ≤ 0.05) in both genes and proteins expression of all studied genes when compared to that of the diabetic group. 

### 2.7. In Silico Binding of Cichoriin and GLUT4, AMPK, and PI3K Markers

**Figure 4:**
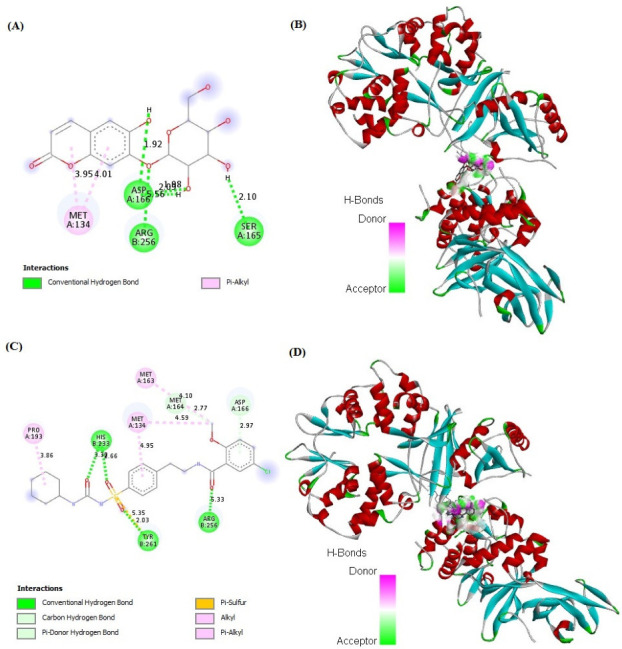
In silico analysis showing docking, amino acid binding interactions and binding pocket of ligand–receptor interactions of cichoriin (**A**,**B**) and GB (**C**,**D**) with GLUT4. Glibenclamide (GB); glucose transporter type 4 (GLUT4).

**Figure 5:**
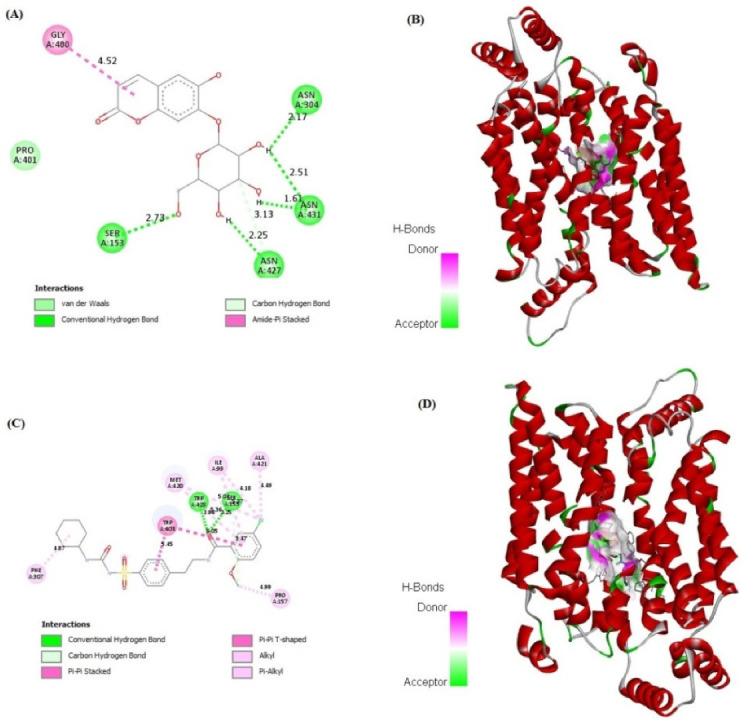
In silico analysis showing docking, amino acid binding interactions and binding pocket of ligand–receptor interactions of cichoriin (**A**,**B**) and GB (**C**,**D**) with AMPK. Glibenclamide (GB); AMP-activated protein kinase (AMPK).

**Figure 6:**
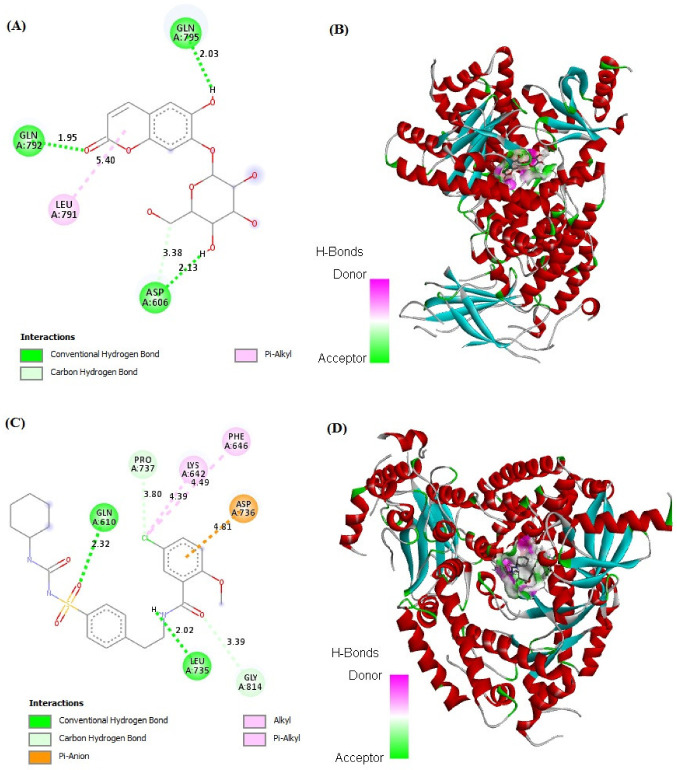
In silico analysis showing (docking, amino acid binding interactions and binding pocket of ligand–receptor interactions) of cichoriin (**A**,**B**) and GB (**C**,**D**) with PI3K. Glibenclamide (GB); phosphatidylinositol 3-kinase (PI3K).

Cichoriin showed strong binding affinity to GLUT4, AMPK, and PI3K markers (−7.48, −4.75 and −6.50 kcal/mol, respectively) in comparison to those of GB binding affinity (−7.49, −5.59 and −7.37 kcal/mol, respectively). The receptors demonstrated a stable interaction with cichoriin via the formation of various bindings. Remarkably, cichoriin formed the following interactions: Conventional hydrogen and Pi–alkyl interactions with GLUT4 (Figure 4A,B), van der Waals, conventional hydrogen, carbon hydrogen and amid –Pi stacked interactions with AMPK (Figure 5A,B) and conventional hydrogen, carbon hydrogen and Pi-alkyl interactions with PI3K (Figure 6A,B). On the other hand, GB developed conventional hydrogen, carbon hydrogen, Pi-donor hydrogen, Pi-sulfur alkyl and Pi–alkyl interactions with GLUT4 (Figure 4C,D), conventional hydrogen, carbon hydrogen, Pi-Pi T-shaped, Pi-Pi stacked, alkyl and Pi–alkyl interactions with AMPK (Figure 5C,D) and conventional hydrogen, carbon hydrogen, alkyl, Pi–alkyl and Pi–anion interactions with PI3K (Figure 6C,D). These findings reveal that cichoriin is a highly competitive agonist of GLUT4, AMPK, and PI3K markers compared to GB.

## 3. Discussion

Many patients with type 2 DM have a resistance to insulin-mediated glucose disposal that is highly associated with impaired insulin secretion [30,31]. Based on previous studies, HFD is considered to be a well-established model for inducing insulin resistance through the accumulation of lipids in various tissues [32]. A low dose of STZ is reported to selectively destroy the pancreatic islet beta-cells, causing mild insulin deficiency [33]. Reports mentioned that the model of combination of HFD with a low dose of STZ potentially induces elevated BGL and mimics the characterizations of human type 2 DM [34]. The current antidiabetic drugs are designed to alleviate DM and its associated complications, but they are costly and demonstrate remarkable adverse side effects. Phytoconstituents have traditionally emerged as a treatment for DM, particularly as they are inexpensive and have a good reputation for presenting fewer side effects [35]. In this context, there is a necessity to explore and develop new natural antidiabetic candidates. Previous records indicated the therapeutic effects of coumarins and their derivatives on diabetes; fraxetin significantly decreased BGL as well as glycosylated hemoglobin and increased the plasma insulin level at a dose of 80 mg/kg body weight [36] and octanoyllomatin exhibited a prominent α-glucosidase inhibition with IC50 value (69 µg/mL) [37]. Further examples include peucedanol glucoside, which showed a 39% reduction in postprandial hyperglycemia at a dose of 5.8 mg/kg [38], and umbelliferone, which revealed a notable antihyperglycemic effect comparable to GB at a dose of 30 mg/kg body weight [39]. The current study was conducted as a thorough evaluation of the possible antidiabetic activity of cichoriin on HFD/STZ-induced diabetic rats. HFD/STZ-induced diabetic rats (group II) denoted marked elevation on BGL and a reduction in serum insulin when compared to group I (control). Histopathological degeneration of pancreatic islets along with inflammation shown in group II also supported the destructive effect of STZ. Additionally, the immunoexpression of pancreatic insulin was significantly inhibited in the diabetic group compared to the control, demonstrating beta-cell dysfunction. Furthermore, HFD/STZ-induced diabetic rats (group II) showed a signification increase in the lipid profile, serum TC and TG compared to the control. HFD caused insulin resistance, rendering glucose uptake by peripheral tissues [38]. The findings of the present study are consistent with previous studies, proving the effectiveness of using both STZ and HFD to induce type 2 DM [33,34]. However, the results (Table 1) revealed that cichoriin (50 and 100 mg/kg) significantly lowered BGL and increased the release of insulin, as well as significantly improving the lipid profile indices in diabetic rats, which was realized from the decreased levels of TC and TG compared to the diabetic group and standard group (5 mg/kg, GB). The findings indicate that cichoriin has the potential to ameliorate the diabetic conditions. Similar to previously mentioned antidiabetic coumarins, isofraxidin, as a coumarin compound, also reduced the elevated TC and TG induced in high-fat feeding rats through the inhibition of lipid production and subsequent inflammation [40]. Furthermore, histopathology examination of the structure of the pancreas demonstrated that treatment with cichoriin, especially high dose (100 mg/kg), reduced the destructive effect of HFD/STZ on pancreatic islets of diabetic rats (Figure 1). The interruption of the pancreatic cell architecture was restored and normalized in the cichoriin (100 mg/kg)-treated diabetic group compared to the untreated diabetic group and standard group (5 mg/kg GB). This protective effect could lead to an improved release of insulin and accordingly ameliorate glucose uptake by tissues, which was seen in a subsequent reduction in BGL (Table 1). The result is congruent with reported studies of Scopoletin (a coumarin compound) with a beneficial effect against pancreas damage [41]. The immuno-histochemical findings presented in the current study demonstrated that cichoriin (50 and 100 mg/kg) can induce significant improvements in the functioning of beta-cells (Figure 2), which was evidenced by the elevated serum insulin level (Table 1). Elevated levels of BG, TC, and TG, as well as the disturbance in levels of oxidative stress markers, including, lipid peroxidation product (MDA) and antioxidant SOD, are often concomitant with the development of DM [30]. In this study, TAC, catalase and SOD levels were markedly decreased and MDA was increased in the HFD/STZ group. Significantly, this oxidative stress was reduced by the treatments with cichoriin (50 and 100 mg/kg) (Table 2). Besides the antioxidant and insulin secretagogue activities of cichorin, it also promotes the peripheral cellular uptake of glucose. The findings of mRNA and protein expressions in treated and diseased groups compared to the control and standard groups showed that cichoriin at both doses (50 and 100 mg/kg) upregulated GLUT4, AMPK, and PI3K in skeletal muscle when compared to that of the diabetic group (Figure 3). Therefore, cichoriin possibly activates the insulin-sensitizing signaling pathway in HFD/STZ-induced stress via a modulatory effect on GLUT4, AMPK, and PI3K biomarkers. The findings are consistent with the results of previous studies on the ameliorative effect of various types of coumarins, including scopoletin, esculin, fraxetin and osthole on such biomarkers in an HFD/STZ-induced diabetic model [42,43]. However, in silico results revealed that both cichoriin and GB bind to GLUT4, AMPK, and PI3K by binding to various amino acid residues (Figure 4, Figure 5 and Figure 6), indicating their possible role in regulating the skeletal-muscle glucose uptake via the activation of insulin-sensitizing signaling pathway of those markers. Taken together, the present findings clearly indicate that cichoriin has the potential to alleviate the HFD/STZ-induced diabetic conditions via its antioxidant effect, improved BGL and lipid profiles, and restored the pancreatic tissue architecture. Consequently, cichoriin could possibly be utilized to produce new antidiabetic medication.

## 4. Materials and Methods

### 4.1. Experimental Animals

Twenty-five adult male Wistar rats (the body weight 200 ± 20 g, aged 9 to 10 weeks old) were procured from the NRC (Giza, Egypt) and maintained to adapt for two weeks before the study. The rats were housed in cages with free access to food and water, at a temperature of 26 ± 2 °C and a 12 h light/dark cycle. The study was approved by the research ethics committee at the Faculty of Pharmacy, Minia University, Egypt (approval number: ES24/2020). The rats were randomly divided to control rats that were fed with a commercially normal pellet diet (NPD) (12% kilocalories as fat), and other rats were used for the induction of type 2 diabetes and then divided according to the treatment applied.

### 4.2. Induction of Type 2 DM

Type 2 DM was induced in rats by a combination of HFD followed by a low dose of STZ (Sigma-Aldrich, St. Louis, MO, USA) according to the previous method with minor modification [34]. Rats were fed with prepared HFD (58% kilocalories as fat; diet components in grams: 365 g NPD, 313 g beef fat, 250 g casein, 60 g mixture of vitamins and mineral, 10 g of cholesterol, 1 g of NaCl, and 1 g of dried yeast per kg). After a period of 3 weeks, HFD-treated rats were fasted overnight and then injected intraperitoneally with STZ (35 mg/kg body weight, freshly dissolved in 0.1 M ice-cold citrate buffer, pH 4.5) [44]. The tail blood glucose levels were measured after 3 days of STZ injection. Rats that had a BGL above 200 mg/dL were considered diabetic and continued to feed on HFD until the end of the experiment [45].

### 4.3. Design of Study

The diabetic rats with the control rats were randomly assigned into five groups (*n* = 5): group I: control, group II: diabetic, groups: III and IV: diabetic rats treated with cichoriin (50 or 100 mg/kg, respectively) (Sigma-Aldrich, St. Louis, MO, USA) [46] dissolved in 1% carboxy methyl cellulose (CMC), respectively), and group V: diabetic rats treated with GB (5 mg/kg) [47] dissolved in 1% CMC (Sigma-Aldrich, St. Louis, MO, USA) and act as a reference diabetic group. Treatment was administrated orally for 14 days. Then, blood samples from the tails were collected after overnight fasting and were used for the measurement of BGL. Rats were decapitated under anesthesia using urethane (1 g/kg, i.p.); blood samples were collected for the separation of serum by centrifugation (3000 rpm, 15 min) and were used to determine biochemical analysis. The pancreas of each rat was taken for histopathological evaluation and kept in 10% formalin. The hepatic and skeletal muscle tissues were flash frozen in liquid nitrogen after being washed in cold saline and dried on filter paper. Afterwards, tissues were kept at −80 °C for further assessment.

### 4.4. Measurement of Blood Glucose Level, Serum Triglycerides, Total Cholesterol and Insulin

Measurement BGL was valued using test strips (Accu-chek active, Roche Diagnostics GmbH, Mannheim, Germany). TG and TC were evaluated using an automated chemistry analyzer (Merck, Wiesbaden, Germany). The concentration of serum insulin was assessed following the instructions of the rat insulin ELISA assay kit (Cusabio, Houston, TX, USA) [45].

### 4.5. Measurement of Antioxidant and Oxidative Stress Parameters

TAC was evaluated in serum in line with the method of Koracevic et al. [48] following the manufacturer’s instructions for the TAC assay kit (Abcam, Cambridge, MA, USA). The activity of SOD and content of MDA were assessed in (20% *w*/*v*) hepatic tissue homogenates according to previously described methods [49,50], respectively. Catalase activity was measured according to the instructions of the commercial kit [51] (Biodiagnostic, Giza, Egypt).

### 4.6. Histopathological Analysis of Pancreatic Tissue 

The collected pancreas tissues were processed to obtain paraffin blocks. Sections at a thickness of 4–5 µm were cut using a microtome (Leica 2025, Microsystems Nussloch GmbH, Nußloch, Germany) and stained with a hematoxylin and eosin (H and E) staining kit (Abcam, ab245880, Cambridge, MA, USA) following standard procedures according to Culling, C.F.A. 2013 [52]. The examination was carried out using a light microscope (Leica Microsystems, GmbH, Germany) at a high magnification of 400×.

### 4.7. Immunohistochemistry of Insulin in Pancreas

Immunohistochemical analysis of insulin expression was performed using insulin polyclonal antibody (1:200) (Bioss Antibodies Inc., Woburn, MA, USA) following the manufacturer’s protocol. Five-micron-thick paraffin-embedded pancreatic-tissue sections were deparaffinized, retrieved, and incubated in 3% H_2_O_2_ for 20 min. Incubation with primary antibody occurred for 1 h at 37 °C after washing with PBS. The incubation with a secondary antibody horseradish peroxidase (HRP) Envision kit (Dako North America, Carpinteria, CA, USA) was performed for 20 min with subsequent washing by PBS. Then, sections were incubated with diaminobenzidine for 15 min and washed with PBS. Sections were counterstained with hematoxylin, then dehydrated and cleared in xylene. Microscopic examination was carried out using an HD imaging system (Leica Microsystems, GmbH, Germany). The mean optical density of the immunohistochemical expression levels of insulin in the stained sections was calculated from six non-overlapping randomly selected fields [53,54].

### 4.8. RT-PCR for GLUT4, AMPK, and PI3K in Skeletal Muscle

RNA was extracted by using a nucleic acid extraction kit (Nucleospin, Macherey-Nagel GmbH & Co. Düren, Germany). The concentrations of the RNA samples and purity (A_260_/A_280_ ratio) were determined via spectrophotometry. The extracted RNA samples were stored at −80 °C until use. cDNA synthesis and real-time PCR were performed using a SensiFAST™ SYBR^®^ Hi-ROX One-Step Kit (Bioline, London, UK). The prepared reaction medium was applied in real-time PCR (Step One Applied Biosystem, Foster city, CA, USA). The primer sequences for the studied targets GLUT4, AMPK and PI3K and reference housekeeping gene glyceraldehyde 3-phosphate dehydrogenase (GAPDH) (Invitrogen, Carlsbad, CA, USA) are shown in Table 3. The data are expressed as the cycle threshold (Ct). The relative quantitation (RQ) of each target gene was performed in accordance with the calculation of delta–delta Ct (ΔΔCt). The RQ of each gene was calculated by performing 2-∆∆Ct normalization to GAPDH [55].

### 4.9. Western Blot for GLUT4, AMPK, and PI3K in Skeletal Muscle

Rabbit polyclonal antibodies for GLUT4 (Protein Tech, Rosemont, IL, USA), AMPK (Cell Signaling Technology, Danvers, MA, USA) and mouse monoclonal PI3K (Santa Cruz Biotechnology, CA, USA) were used. The total proteins were extracted from skeletal muscle homogenates using a readyprep^TM^ protein extraction kit (Bio-Rad, CA, USA). The quantification of protein was performed using a Bradford protein assay kit. An equal protein sample of 20 μg was loaded with an equal volume of 2× Laemmli sample buffer containing 0.125 M Tris HCl (pH 6.8), 20% glycerol, 4% SDS, 10% 2-mercaptoehtanol, and 0.004% bromophenol blue. The protein was denatured by boiling at 95 °C for 5 min and then separated by 10% SDS-PAGE and transferred onto PVDF membranes using BioRad Trans-Blot Turbo. The blotting buffer is composed of 25 mM Tris and 190 mM glycine and 20% methanol. The membranes were blocked with TBST buffer (consisting of 20 mM Tris pH 7.5, 150 mM NaCl, 0.1% tween 20) and 3% bovine serum albumin. The blocking step was performed at room temperature for 1 h. After that, overnight incubation with the primary antibodies at 4 °C was carried out. The membranes were incubated with HRP-conjugated secondary antibodies at room temperature for 1 h. The target protein bands were visualized using chemiluminescent substrate kit (Bio-Rad, CA, USA) on the ChemiDoc MP imager (Invitrogen, Carlsbad, CA, USA). The intensity of target protein bands was analyzed against beta actin (housekeeping protein) using the ImageJ software [56].

### 4.10. Computational Analysis

The docking assessment was conducted using the programs of docking as described earlier by Khalil et al. [50]. The PubChem database was the available source for retrieving the structure of ligands. Computational studies were performed to explicate the molecular interactions of cichoriin (CID-442101) and GB (CID-3488) ligands. The crystal structures of the mice AMPK (PDBID: 5ufu), PI3K (PDB ID: 7r2b) and GULT4 (PDBID: 7wsn) were retrieved from the Protein Data Bank (https://www.rcsb.org/) (accessed on 10 August 2022). Docked ligand–receptor interactions were visualized and analyzed using Discovery Studio 2021 Client trial version, as described earlier by Khalil et al. [57].

### 4.11. Statistical Analysis

The obtained results were shown as mean ± standard error (S.E). The analysis was performed using Graph Pad Prism (software V 8.2, San Diego, CA, USA). One-way ANOVA was used to assess the comparisons between the control and treatment groups, while Tukey’s multiple comparisons test was employed to check differences among the groups. Differences were considered significant at *p* ≤ 0.05.

## 5. Conclusions

In brief, the present study demonstrated the possible mechanistic role of cichoriin as an ameliorative candidate in HFD/STZ-induced diabetic conditions. The results showed that cichoriin treatments mitigated the diabetic symptoms via a reduction in BGL, TC, TG and MDA, in addition to improving SOD. Furthermore, histopathological evaluation revealed the restoration of pancreatic tissue architecture and function following the treatment with cichoriin. The research work revealed the modulatory effect of cichoriin on GLUT4, AMPK, and PI3K biomarkers, indicating its possible role in the regulation of glucose uptake. The in silico investigation supported the possible binding and interaction of cichoriin with biomarkers. Future clinical studies on cichoriin are recommended to establish its potential as a new therapeutic and protective tool against diabetes.

## Figures and Tables

**Table 1:** Effect of cichoriin on biochemical parameters in rats.

Groups	Group I (Control)	Group II (Diabetic)	Group III (50 mg/kg, Cichoriin)	Group IV (100 mg/kg, Cichoriin)	Group V (5 mg/kg, GB)
BGL (mg/dL)	103 ± 4	452 ± 44 ^a^	206 ± 7 ^a,^^b^	127 ± 3 ^b^	128 ± 5 ^b^
Serum Insulin (nIU/mL)	7.8 ± 0.2	2.6 ± 0.1 ^a^	4.8 ± 0.2 ^a,b^	7.0 ± 0.2 ^a,b,c^	7.2 ± 0.1 ^b,c^
TG (mg/dL)	64.7 ± 9.7	127.7 ± 19.4 ^a^	99.5 ± 4.8	76.9 ± 9.2 ^b^	81.9 ± 5.6
TC (mg/dL)	65.4 ± 5.5	99.5 ± 5.6 ^a^	83.8 ± 2.1	78.0 ± 4.9 ^b^	77.6 ± 4.4 ^b^

Data were presented as means ± SE (*n* = 5). ^a^, ^b^, and ^c^ indicate significant difference from group I, group II, and group III, respectively, at (*p* *≤* 0.05). Glibenclamide (GB); Blood glucose level (BGL); Triglyceride (TG); Total cholesterol (TC).

**Table 2:** Effect of cichoriin on oxidative stress markers.

	Group I (Control)	Group II (Diabetic)	Group III (50 mg/kg, Cichoriin)	Group IV (100 mg/kg, Cichoriin)	Group V (5 mg/kg, GB)
TAC (mM/L)	1.70 ± 0.01	0.60 ± 0.05 ^a^	0.75 ± 0.03 ^a^	0.79 ± 0.06 ^a,b^	0.83 ± 0.04 ^a,b^
Catalase (U/L)	371.4 ± 31.5	151.0 ± 24.9 ^a^	217.5 ± 15.7 ^a^	395.9 ± 18.5 ^b,c^	429.9 ± 29.2 ^b,c^
SOD (U/g tissue)	1476 ± 64.4	1236 ± 32.1 ^a^	1314 ± 34.0	1526 ± 25.2 ^b,c^	1554 ± 17.2 ^b,c^
MDA (nmol/g tissue)	39.4 ± 1.9	64.0 ± 5.7 ^a^	53.04 ± 3.8	38.7 ± 1.5 ^b,c^	37.8 ± 1.7 ^b,c^

Data were presented as means ± SE (*n* = 5). ^a^, ^b^, and ^c^ indicate significant difference from group I, group II, and group III, respectively, at (*p* ≤ 0.05). Glibenclamide (GB); Total antioxidant capacity (TAC); superoxide dismutase (SOD); malondialdehyde (MDA).

**Table 3:** Primer sequences of all the studied genes.

Gene Symbol	Primer Sequence From 5′–3′
GLUT 4	F: GGTTCCATCCATGAGAGTTATGTGTC R: CTAAAGAGAGAAGGTGTCCGTCG
AMPK	F: GTGCCTATGAGCACCAAGTCAG R: TTCATGCTCTGGTTAGGGTGAG
PI3K	F: TTAAACGCGAAGGCAACGA R: CAGTCTCCTCCTGCTGTCGAT
GAPDH	F: CCTCGTCTCATAGACAAGATGGT R: GGGTAGAGTCATACTGGAACATG

Glucose transporter type 4 (GLUT4), AMP-activated protein kinase (AMPK) and phosphatidylinositol 3-kinase (PI3K) and glyceraldehyde 3-phosphate dehydrogenase (GAPDH).

## Data Availability

The data presented in this study are available on request from the corresponding author.

## Sample Availability

Sample of cichoriin is available from the authors.
